# The neurohypophyseal hormone oxytocin and eating behaviors: a narrative review

**DOI:** 10.1007/s42000-023-00505-y

**Published:** 2023-11-18

**Authors:** Michele Iovino, Tullio Messana, Simonetta Marucci, Domenico Triggiani, Vito Angelo Giagulli, Edoardo Guastamacchia, Giuseppina Piazzolla, Giovanni De Pergola, Giuseppe Lisco, Vincenzo Triggiani

**Affiliations:** 1 Interdisciplinary Department of Medicine, Section of Internal Medicine, Geriatrics, Endocrinology and Rare Diseases, University of Bari “Aldo Moro”, School of Medicine, Bari, Apulia Italy; 2 Infantile Neuropsychiatry, IRCCS – Institute of Neurological Sciences, Bologna, Italy; 3 Università Campus Biomedico, Dip. “Scienze e Tecnologie per l’Uomo e l’ambiente”, Via Alvaro del Portillo, 21 Roma, Italy; 4 National Institute of Gastroenterology IRCCS “Saverio de Bellis”, Research Hospital, Castellana Grotte, Bari, Italy; 5 Department of Biomedical Science and Human Oncology, University of Bari, School of Medicine, Bari, Apulia Italy

**Keywords:** Oxytocin, Oxytocin receptor, Food intake, Eating behaviors, Eating disorder, Obesity, Disordered eating

## Abstract

**Background:**

The neuropeptide oxytocin (OT) is crucial in several conditions, such as lactation, parturition, mother-infant interaction, and psychosocial function. Moreover, OT may be involved in the regulation of eating behaviors.

**Methods:**

This review briefly summarizes data concerning the role of OT in eating behaviors. Appropriate keywords and medical subject headings were identified and searched for in PubMed/MEDLINE. References of original articles and reviews were screened, examined, and selected.

**Results:**

Hypothalamic OT-secreting neurons project to different cerebral areas controlling eating behaviors, such as the amygdala, area postrema, nucleus of the solitary tract, and dorsal motor nucleus of the vagus nerve. Intracerebral/ventricular OT administration decreases food intake and body weight in wild and genetically obese rats. OT may alter food intake and the quality of meals, especially carbohydrates and sweets, in humans.

**Discussion:**

OT may play a role in the pathophysiology of eating disorders with potential therapeutic perspectives. In obese patients and those with certain eating disorders, such as bulimia nervosa or binge/compulsive eating, OT may reduce appetite and caloric consumption. Conversely, OT administered to patients with anorexia nervosa may paradoxically stimulate appetite, possibly by lowering anxiety which usually complicates the management of these patients. Nevertheless, OT administration (e.g., intranasal route) is not always associated with clinical benefit, probably because intranasally administered OT fails to achieve therapeutic intracerebral levels of the hormone.

**Conclusion:**

OT administration could play a therapeutic role in managing eating disorders and disordered eating. However, specific studies are needed to clarify this issue with regard to dose-finding and route and administration time.

## Background

Hypothalamic nuclei play a role in eating behaviors, stimulating or inhibiting food intake. Appetite is triggered by nuclei located in the lateral hypothalamus, also known as the “hunger center,” while it is suppressed by neurons located in the ventromedial nucleus of the hypothalamus (VMH) (“satiety center”) [1–5].

Oxytocin (OT), a neurohypophyseal hormone, is synthesized by hypothalamic magnocellular neurosecretory neurons (MCN) of the supraoptic (SON) and paraventricular nuclei neurons (PVN) [6, 7]. It is pivotal in inducing parturition and lactation and in regulating social behaviors. Also, OT is synthesized in other areas of the forebrain, such as the amygdala, hippocampus, septum, striatum, and bed nucleus of the terminal stria and is also detectable in the cerebrospinal fluid [6, 7].

**Fig. 1:**
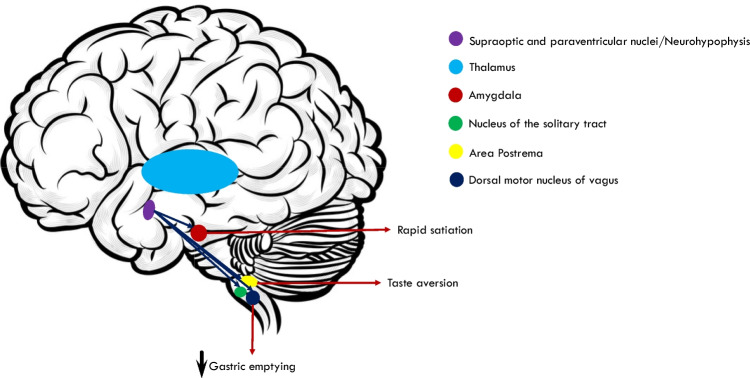
Simplified depiction of the relationship between OT-ergic hypothalamic PVN and SON neurons and brain sites involved in regulating eating behaviors (blue arrows). OT-ergic projections from PVN synapse within the amygdala (AMY), area postrema (AP), nucleus of the solitary tract (NTS), and dorsal motor nucleus of vagus nerve (DMV) playing a role in anorexigenic response, taste aversion, gastric emptying (red arrows)

The role of OT in regulating eating behaviors has yet to be understood entirely. In this review, we have summarized the neuroanatomical and biochemical OT-ergic pathways synapsing on brain areas involved in regulating eating behaviors in animal models and humans (Fig. 1). PubMed/MEDLINE was searched for references of original articles and reviews. Appropriate keywords and medical subject headings terms were identified and included the following: “oxytocin,” “eating disorders,” “eating behaviors,” “hypothalamus,” “hypothalamic ventromedial nucleus,” “amygdale,” “area postrema,” “nucleus of the solitary tract,” and “dorsal motor nucleus of the vagus nerve.” References were screened according to a hierarchical strategy by title and abstract, and full text. Original papers and reviews were screened, selected when appropriate, and discussed in detail.

## Overview of physiological aspects of oxytocin

The stimulation of the cervix uteri and nipples stimulates the synthesis of a precursor protein encoded by the OT gene to synthesize OT and its carrier protein neurophysin 1 [8, 9]. These peripheral signals originating from cervix uteri and nipple receptors are transmitted to the SON and PVN OT-ergic neurons via the spinal cord, the dorsal lateral fasciculus, the medial forebrain bundle, and the mammillary peduncle [10–12]. The glial cells, mainly astrocytes, stimulate the morphological plasticity of the OT-ergic neurons during lactation and parturition to facilitate the synthesis and transport of OT [10, 13].

The neurokinin 3 receptor is associated with the nuclear chaperone protein importin β1, which induces the internalization of the OT precursor in the Golgi complex and rough endoplasmic reticulum [10, 11].

Once synthesis has occurred, OT and its carrier protein neurophysin 1 are conveyed to the neurohypophysis where they are stored in secretory vesicles and released into the systemic circulation [10, 13, 14].

The OT receptor (OTR) is a Gq/11 protein-linked receptor, and OT-binding sites have been localized in the brain. Efferent pathways arising from the hypothalamic OT-ergic neurons project to brain areas containing a high concentration of OTR, including the olfactory bulb, bed nucleus of the stria terminalis, nucleus accumbens septi, hypothalamic suprachiasmatic, arcuate and VM nuclei, amygdala, hippocampus, septum, the nucleus of the solitary tract (NTS) of the oblongata medulla, cingulate cortex, and spinal cord [15]. OTR is also expressed in peripheral tissue such as the kidney, heart, thymus, pancreas, and adipose tissue [16].

## Neuroanatomical organization of regions regulating food intake, appetite, satiety, and reward

Hypothalamic OT-ergic neurons display widespread projections throughout the brain, mainly to the amygdala, contributing to the satiety process by causing the sensation of fullness [17]. Other brain areas typically regulating eating behaviors receive afferent projections from hypothalamic OT-ergic neurons, including the area postrema, the NTS, and the dorsal motor nucleus of the vagus nerve [18, 19].

### The ventromedial nucleus of the hypothalamus

The VMH, the Cajal nucleus, is a pear-shaped structure in the hypothalamic tuberal area. The neurons can synthesize OTR and express a high density of OTR on their surfaces [20, 21]. Food intake is inhibited, weight gain restricted, and energy expenditure augmented after the leptin-induced activation of steroidogenic factor 1 positive neurons in the VMH [22, 23].

Food intake, especially fasting-absorbed carbohydrates, stimulates OT release from OT-secreting neurons [24]. Moreover, several hormones may stimulate OT release after the ingestion of a meal, including leptin, cholecystokinin, and gastrointestinal incretins. The same is observed after noradrenergic stimulation of OT-secreting neurons by vagal afferences from the nucleolus of the solitary tract (NTS) [25].

It has been hypothesized that OT may be involved in regulating food intake and energy expenditure directly or by potentiating central and peripheral anorexigenic stimuli [26].

Although the VMH has a high density of OTR, it contains a few OT-ergic projections, suggesting that it could be a local target of OT [21]. To support this hypothesis, OT antagonism or silencing of the OT-induced signaling pathway in the VMH predisposes to a much-extended food intake in terms of energy intake, delayed satiation, and intake of more carbohydrates while reducing energy expenditure [27–29].

### Extrahypothalamic structures involved in the regulation of eating behaviors

#### The amygdala

Innate appetite and food aversion are modulated by specific brain structures, mainly in the limbic system. The basolateral and central nuclei of the amygdala regulate the appetite in terms of the amount of ingested food and innate aversions and control qualitative predisposition toward specific food due to acquired experience [17]. In rats, the apomorphine administration before or 30 min after the ingestion of saccharine negatively affected further saccharine intake after vomiting. This response indicates that the unpleasant gastric effect of apomorphine significantly contributed to taste aversion, ultimately affecting specific food intake such as rapidly absorbed carbohydrates [30].

The amygdala determines satiation by oropharyngeal and gastric afferents; bilateral amygdala lesions lead to overeating. The suppression of food intake is mediated by cholinergic stimulation of the amygdala. Conversely, adrenergic stimuli enhance appetite and food intake in starving but not satiated animals. Therefore, the amygdala exerts two different influences on eating behaviors. First, the amygdala plays a facilitating effect in the maintenance of consuming activity induced by NA-ergic activation. Subsequently, the amygdala plays an inhibitory role leading to satiety and food intake cessation. This subsequent behavior is due to cholinergic activation, which stops NA-ergic ones [30].

OTRs are expressed on the membrane of the amygdala’s basolateral and central neurons. OT-ergic projections from the PVN nuclei to the amygdala have also been described. Experiencing gastrointestinal toxicity concomitantly to food intake is accompanied by OT release in humans and animals. Therefore, it is believed that OT interaction with cholinergic and adrenergic circuits within the amygdala may have a role in regulating eating behaviors in terms of food intake, satiety, and taste aversion or predilection [31–33]. Indeed, OT administration in the basolateral amygdala effectively suppresses the consumption of palatable saccharin solutions in rats. A moderate restriction of food intake was observed after the administration of OT and was attenuated by pretreatment with an OTR antagonist (L-368,899) [34]. In experimental conditions, assessing the role of OT in mediating the acquisition and retrieval of conditioned taste aversion in mice that underwent lithium-induced acute gastric toxicity, OT was found to contribute to causing taste aversion significantly. At the same time, OT antagonism partially alleviated it but did not wholly retrieve taste aversion [35].

#### The area postrema

Lesions of the medullary circumventricular organ in the area postrema (AP) reduce food intake and induce weight loss [36, 37]. As a chemo-sensitive organ, AP modulates the conditioned avoidance response (CAR) to toxins, such as carbonate lithium. The acquisition of CAR and conditioned palatability to oral sucrose was assessed in rats with lesions of the AP. The abolishment of OT-ergic inputs from the hypothalamic PVN induced restricted ingestion and increased aversive responses to intraoral infusion of sucrose following an intraperitoneal injection of carbonate lithium [38].

OT was found to increase intragastric pressure by vagal efferences after its administration in the fourth ventricle, specifically acting at the level of AP and NTS [39]. The peptide hormone amylin, or islet amyloid peptide, is co-secreted with insulin from the pancreatic β-cells and promotes satiety by decelerating gastric emptying. The precise mechanism by which amylin reduces food intake is mediated by the activation of NA-ergic neurons within the AP [40]. Similar effects are also induced by calcitonin, a potent amylin agonist structurally similar to amylin, as belonging to the calcitonin-like gene peptide superfamily. AP lesions affecting OT-ergic release abolish anorexic effects induced by the peripheral administration of amylin and calcitonin [41]. This could be an additive mechanism by which OT may reduce food intake and prompt satiety.

#### The nucleus of the solitary tract and the dorsal motor nucleus of the vagus nerve

The NTS of the dorsal medulla oblongata plays an essential role in regulating cardiovascular functions, affects the activity of hypothalamic SON and PVN neurons [42, 43], and reduces food intake and body weight [44]. Signals from the gastrointestinal tract are conveyed to the brain by vagal afferents synapsing within the medullary dorsal vagal complex, including the NTS and the dorsal motor nucleus of the vagus nerve (DMV) [45, 46]. Moreover, descending pathways from the hypothalamic SON and PVN to the NTS and DMV are involved in the beginning and termination of food intake [47].

An experimental injury of the median-caudal region of the NTS induced hypophagia with consequent body weight loss [48]. During the first 6 days following the electrolytic lesion of the NTS, rats reduced their food intake by around 80% compared to the sham controls. From the 7th day, food intake slightly recovered, but the appetite remained significantly reduced compared to baseline [37].

OT may regulate appetite, food intake, and weight gain by acting at the NTS site. An experiment in rats suggested that the administration of OT in the NTS decreased caloric intake by reducing food motivation and seeking [49]. In rats, acute intraventricular administration of OT (5 μg) 30 min before a meal consumption induced a dose-response reduction in food intake up to 72% (3rd ventricle) and 60% (4th ventricle). Chronic exposure to OT prevented excessive weight gain after exposing the rats to overfeeding with a high-fat diet, with OT-treated animals maintaining a higher leptin sensibility than vehicle controls [50].

Glucagon-like peptide 1 (GLP-1) inhibits food intake by acting on neurons within the NTS and abolishes food reward behaviors and motivation to food intake. Microinjections of native GLP-1 or the GLP-1 analog exendin-4 into the NTS suppressed food reward behaviors, thus reducing appetite and food consumption and, lastly, leading to weight loss. These effects are related to the food reward-suppressing role of GLP-1 agonists operating within the NTS [51].

Adrenalectomy reduces food intake significantly, but this response is reversed by OTR antagonists and by activating satiety-related responses in the NTS. It has been reported that OT-ergic projections from the PVN to NTS are highly upregulated after bilateral adrenalectomy, thus positively affecting satiety and consequently reducing meal size in primary adrenal insufficiency [52]. Glucocorticoid replacement therapy prompts the opposite effect.

Evidence shows that OT directly injected in the dorsal vagal complex (DVC) stimulates gastric secretion via the vagal pathway [53]. OT levels in the DVC were significantly increased in response to food intake, and OTR signaling within DVC neurons plays a counter regulator of gastrointestinal activity by stimulating satiation signals to reduce food intake [54].

## Oxytocin and eating behaviors: what do we know?

OT may have a role in controlling emotion and cognition [55] and regulating food intake [56]. In normal-weight and obese animals, OT administered centrally reduces food intake and facilitates weight loss [57]. These responses were also observed when OT was injected peripherally [58]. Moreover, a pretreatment with both central and peripheral (fully permeable to the blood-brain barrier) administration of OTR antagonists reduced the attenuation of food intake after OT administration [57, 58].

Rats with lesions of the PVN, the leading site of OT secretion, exhibited more food intake and weight gain than controls [59]. In this model, the peripheral and central administration of OT reduced, in a dose-dependent manner, food intake and increased the time intercurrent of the consumption of two consecutive meals [60]. The results suggest that OT could be involved in the induction and prolongation of satiety.

Patients with neuropsychiatric diseases often exhibit eating disorders, with hyperphagia and increased meal size usually the leading determinant of weight gain. The therapeutic potential of OT was analyzed in this cluster of patients, hypothesizing that OT could have also played a role in the regulation of psychosocial functions coupled with eating behaviors. A pivotal study conducted in 16 patients with an established diagnosis of schizophrenia on a stable antipsychotic treatment with overweight or obesity (BMI > 27 kg/m^2^) showed that the intranasal administration of OT (24 IU) compared to placebo a few minutes before the meal consumption did not affect satiety, meal size, post-meal serum glucose, and insulin levels [61].

In a randomized clinical trial, administering OT twice daily for 3 months compared to placebo improved social behavior and reduced appetite in children (3–11 years) with Prader-Willy syndrome [62].

The encephalic functional magnetic resonance imaging revealed that the intranasal administration of OT in obese men attenuated the ventral tegmental area firing to food motivation regions such as the insula, oral somatosensory cortex, and amygdala in response to high-calorie visual food images. The results suggested that OT may exert an anorexigenic effect by dampening eating cravings activated by reward anticipation in patients with obesity [28].

In women with stress-induced eating disorders, the overall exposure to serum cortisol is usually higher than normal. This mechanism may contribute to increase the appetite and positively affect food intake. The intranasal administration of a 24 IU shot compared to the placebo (saline solution) was found to reduce the intake of sweet and fatty snakes by 15 min after administering the neurohypophyseal hormone. Interestingly, the salivary cortisol levels (assessed to test the level of stress) throughout the observation remained unchanged up to 75 min after the administration of OT [63]. The findings were consistent with the fact that OT affected eating behavior independently of the background stress level by acting with a direct mechanism.

To better investigate the efficacy, safety, and mechanisms via which OT is involved in reducing appetite, caloric intake, and body weight and affecting energy expenditure, body composition, glucose and lipid metabolism, and brain activation and control of behaviors and impulses in response to food images, an 8-week randomized, double-blind, placebo-controlled trial has been designed and is currently ongoing. The study will clarify several exciting issues about OT as a pharmacologic treatment of obesity [64]. Moreover, dysfunction in the OT-ergic mechanisms has also been reported in patients with anorexia nervosa, with specific patterns that include lower circulating levels of OT at fasting and after stimulation, lower nocturnal levels of OT, and higher peripheral OT concentration after meal ingestion [65]. Derangements of OT homeostasis in AN are close to the opposite of those observed in other eating disorders characterized by weight excess or propension to gain weight and are reversible after rehabilitation programs and weight gain in AN. Although no specific trials have been carried out, OT administration may have a particular and disease-related role in improving food intake in AN. The mechanisms potentially explaining this sui generis and paradoxical effect could be attributable to the contribution of OT administration in reducing eating-related attention and concerns, attenuating cognitive rigidity, improving emotional expression, and weakening the attitude of avoiding social situations or contexts emotionally provoking stimuli, lastly improving social behavior [66, 67].

The observation of a dimorphic action of OT in these two kinds of eating disorders suggests that OT regulates the brain circuits subserving eating behaviors. Therefore, the physiological involvement of OT in eating disorders can support its beneficial therapeutic effect in clinical practice since the administration of OT by intranasal route may bypass the blood-brain barrier [68] and reach the amygdala and brainstem structures involved in the control of eating behaviors such as the AP, NTS, and DMV.

However, the current level of evidence does not suggest a possible positive effect of intranasal OT treatment in eating disorders. This could be attributable to the fact that cerebral exposure to OT, after its intranasal administration, may not be sufficient to elicit desirable effects and probably higher doses, alternative routes, and timing of administration should be considered [69].

Hypothalamic injury has a wide range of etiology, including brain surgery, encephalic trauma, tumors, chemotherapy and radiation, vascular diseases (aneurysms), cerebral infections, and inflammatory and infiltrative diseases. Depending on the sites, a hypothalamic injury may hypothetically result in different clinical consequences [70]. A lesion in the middle hypothalamic region produces direct damage to some specific centers, such as the arcuate nucleus, which is responsible for the tonic release of dopamine, suppressing the prolactin secretion from lactotrophic cells in the pituitary and phasic release of the growth hormone releasing hormone, resulting in a loss of somatotropic cells pulses of growth hormone. Moreover, the dorsomedial and ventromedial nuclei are also located in the middle region of the hypothalamus and are directly involved in controlling behaviors and gastrointestinal motility (the former) and food intake (the latter). This region may be affected in some endocrine diseases, especially pituitary macro/giant adenomas with considerable suprasellar extension or in the case of primitive hypothalamic disorders (such as craniopharyngioma or infiltrative diseases), and the consequent hypothalamic damage usually results in a progressive deterioration of food intake control, aggressive behaviors, and typically mild or moderate hyperprolactinemia. Injuries in the anterior region of the hypothalamus may harm both the supraoptic and paraventricular nuclei, thus also affecting the OT synthesis [71]. The disturbance may also be accompanied by partial or extensive anterior and posterior pituitary failure, resulting in a unimodal or multimodal hormonal deficiency. Patients with craniopharyngioma, one of the most common causes of hypothalamic damage, usually exhibit lower circulating levels of OT at baseline and after stimulation [72]. Several studies have been published seeking to determine whether OT deficiency was associated with changes in social cognition [73] and eating behaviors in craniopharyngioma survivors. Anecdotal cases suggested that the intranasal administration of OT improved emotional tasks and social behaviors in young survivors of craniopharyngioma with low (case report) [74] and detectable basal levels of OT (case series) [75]. In one cross-sectional case-control study in 34 patients with craniopharyngioma and 73 controls, adverse eating behaviors and eating disorders were more frequently observed among patients with extensive (anterior and posterior) hypothalamic injury than in those with less extensive damage, and controls. Among individuals with adverse eating behaviors, lower postprandial levels of OT compared to control were also found [76], as observed in patients with obesity [71]. The intranasal administration of OT in combination with the opiate antagonist naltrexone (10 weeks of OT alone + 38 weeks of OT and naltrexone) was significantly effective in reducing the appetite, caloric intake, and hyperphagia in a 13-year-old boy with confirmed hypothalamic obesity and hyperphagia post-resection of craniopharyngioma [77]. These positive results could be attributable to the numerous metabolic effects of the neurohormone, including direct reduction of food intake by decreasing appetite in homeostatic and reward-driven conditions (hence, properly insisting on hypothalamic regions involved in the regulation of hunger and satiation), enhancement of lipolysis and energy expenditure, positive affection of body composition due to improvement of peripheral insulin sensitivity, ultimately favoring lean over fat mass building [78]. Nevertheless, a recently published randomized, double-blind, placebo-controlled, crossover pilot study (13 patients randomized; 10 concluded) did not find any relevant changes in body weight between the OT arm 16–24 IU at the three main mealtimes and placebo after 8 weeks of treatment [79]. This finding lays the basis for better-designed multicentric trials to assess the role of OT treatment (alone or in combination) in patients with hypothalamic injuries/dysfunction.

## Discussion and conclusion

One piece of evidence suggests that OT and OTR may regulate eating behaviors and food intake. In neuropsychiatric disorders manifesting with altered eating behaviors, such as anorexia nervosa, OT and OTR agonists could potentially have pharmacological use [80]. In addition, OTR gene polymorphisms may also be involved in the pathogenesis of such disorders [81].

OT administration reduces food intake in patients with bulimia nervosa, thus playing a possible protective effect by limiting food overconsumption, weight gain, and purging behaviors [82].

When OT is administered before meal consumption in healthy individuals, the caloric intake remains unchanged, even if the predilection toward carbohydrates and sweets could be reduced. Conversely, overweight and obese individuals exhibit different responses to OT administration before meals, including caloric restriction, less preference for fatty snake consumption, and unaltered propension toward carbohydrates [83].

Although OT may be enumerated as another therapeutic tool to manage weight gain or induce weight loss [84] and despite anecdotal evidence suggesting that OT administration may improve social behaviors, emotional tasks, and eating behaviors, trials are needed for deeper insight into the therapeutic role of OT in patients with hypothalamic injury, such as craniopharyngioma.

Special studies are surely necessary to verify more precisely the therapeutic role of OT in certain disorders characterized by overeating, eating disorders, and disordered eating as well.

## Abbreviations

AP: Area postrema
DMV: Dorsal motor nucleus of the vagus nerve
DVC: Dorsal vagal complex
GLP-1: Glucagon-like peptide 1
MCN: Magnocellular neurosecretory neurons
NA: Noradrenaline
NTS: Nucleus of the solitary tract
OT: Oxytocin
OTR: Oxytocin receptor
PVN: Paraventricular nuclei neurons
SON: Supraoptic
VMH: Ventromedial nucleus of the hypothalamus
