# Correction: The neurohypophyseal hormone oxytocin and eating behaviors: a narrative review

**DOI:** 10.1007/s42000-024-00540-3

**Published:** 2024-03-18

**Authors:** Michele Iovino, Tullio Messana, Simonetta Marucci, Domenico Triggiani, Vito Angelo Giagulli, Edoardo Guastamacchia, Giuseppina Piazzolla, Giovanni De Pergola, Giuseppe Lisco, Vincenzo Triggiani

**Affiliations:** 1 Interdisciplinary Department of Medicine, Section of Internal Medicine, Geriatrics, Endocrinology and Rare Diseases, School of Medicine, University of Bari “Aldo Moro”, Bari, Apulia, Italy; 2 Infantile Neuropsychiatry, IRCCS – Institute of Neurological Sciences, Bologna, Italy; 3 Dip. “Scienze e Tecnologie per l’Uomo e l’ambiente”, Università Campus Biomedico, Via Alvaro del Portillo, 21, Roma, Italy; 4 National Institute of Gastroenterology IRCCS “Saverio de Bellis”, Research Hospital, Castellana Grotte, Bari, Italy; 5 Department of Biomedical Science and Human Oncology, School of Medicine, University of Bari, Bari, Apulia, Italy

**Correction: Hormones (2023) 23:15-23**

10.1007/s42000-023-00505-y

In this article the affiliation details for Giovanni De Pergola were incorrectly given as ‘National Institute of Gastroenterology “Saverio de Bellis”, Research Hospital, Castellana Grotte, Bari, Italy’ but should have been ‘National Institute of Gastroenterology IRCCS “Saverio de Bellis”, Research Hospital, Castellana Grotte, Bari, Italy’.

The original article has been corrected.

